# Vacuolar Iron Transporter *BnMEB2* Is Involved in Enhancing Iron Tolerance of *Brassica napus*

**DOI:** 10.3389/fpls.2016.01353

**Published:** 2016-09-13

**Authors:** Wei Zhu, Rong Zuo, Rongfang Zhou, Junyan Huang, Minqiang Tang, Xiaohui Cheng, Yueying Liu, Chaobo Tong, Yang Xiang, Caihua Dong, Shengyi Liu

**Affiliations:** ^1^The Key Laboratory of Biology and Genetic Improvement of Oil Crops, The Ministry of Agriculture of PRC, Oil Crops Research Institute, Chinese Academy of Agriculture SciencesWuhan, China; ^2^Hubei Collaborative Innovation Center for Green Transformation of Bio-Resources, Hubei UniversityWuhan, China; ^3^Guizhou Rapeseed Institute, Guizhou Academy of Agricultural SciencesGuiyang, China

**Keywords:** *Brassica napus*, *BnMEB2*, vacuolar iron transporter, vacuolar sequestration, iron tolerance

## Abstract

Iron toxicity is a nutrient disorder that severely affects crop development and yield in some soil conditions. Vacuolar detoxification of metal stress is an important strategy for plants to survive and adapt to this adverse environment. Vacuolar iron transporter (*VIT*) members are involved in this process and play essential roles in iron storage and transport. In this study, we identified a rapeseed *VIT* gene *BnMEB2* (BnaC07g30170D) homologs to *Arabidopsis MEB2* (At5g24290). Transient expression analysis revealed that BnMEB2 was localized to the vacuolar membrane. Q-PCR detection showed a high expression of *BnMEB2* in mature (60-day-old) leaves and could be obviously induced by exogenous iron stress in both roots and leaves. Over-expressed *BnMEB2* in both *Arabidopsis* wild type and *meb2* mutant seedlings resulted in greatly improved iron tolerability with no significant changes in the expression level of other *VIT* genes. The mutant *meb2* grew slowly and its root hair elongation was inhibited under high iron concentration condition while *BnMEB2* over-expressed transgenic plants of the mutant restored the phenotypes with apparently higher iron storage in roots and dramatically increased iron content in the whole plant. Taken together, these results suggested that *BnMEB2* was a *VIT* gene in rapeseed which was necessary for safe storage and vacuole detoxification function of excess iron to enhance the tolerance of iron toxicity. This research sheds light on a potentially new strategy for attenuating hazardous metal stress from environment and improving iron biofortification in Brassicaceae crops.

## Introduction

Iron is an essential micronutrient for plant growth and human health. It also functions as a required cofactor for various enzymes in DNA biosynthesis and in the electron-transport chains of respiration and photosynthesis (Curie and Briat, 2003; Conte and Walker, 2011). However, tight control of iron acquisition and translocation helps to keep iron homeostasis which is crucial step for all plants survival and proliferation. Iron deficiency in uptake or absorption leads to chlorosis in the young leaves and retarded growth, consequently resulting in reduced photosynthetic efficiency and crop productivity (Wu et al., 2010). In contrast, iron excess can cause severe functional disorders and cellular damages which is highly reactive and toxic (Briat et al., 2010). With the rapid development of modern industry, urban pollution and extensive application of agricultural chemical substances to the soil increased the toxicity of metal pollution which in turn limited the grain production. The breeding of tolerant crop varieties becomes a powerful approach to address the problem of metal toxicity.

Plants have evolved two distinct strategies for iron acquisition (Hindt and Guerinot, 2012; Jain and Connolly, 2013). Dicots and non-graminaceous plants secrete protons into the rhizosphere to enhance the solubility of ferric complex (Fe^3+^), then the root ferric chelate reductase (FRO2) reduces Fe^3+^ (ferric ion) into a more soluble Fe^2+^ (ferrous ion) (Robinson et al., 1999). Finally iron regulated transporter1 (IRT1)-type ferrous transporters move Fe^2+^ across the root epidermal plasma membrane (Vert et al., 2002). Interestingly, the graminaceous plants possess another unique strategy in the uptake of iron. Plants secrete the mugineic acid, a phytosiderophores (PS), into the rhizosphere to form the chelate complex of Fe^3+^-PS (Ishimaru et al., 2006) and then absorb it into root cells by the yellow stripe 1 (YS1) transporter (Curie et al., 2001). Following the initial uptake of iron into root cells, several key genes such as *AtFRD3* (Durrett et al., 2007), *OsFRDL1* (Yokosho et al., 2009), *PEZ1* (Ishimaru et al., 2011), *FPN1* (Morrissey et al., 2009) and *YSL* (Curie et al., 2009; Chu et al., 2010) have been reported to unload and transport iron from root to the above-ground portions of plant such as shoot, leaf and seed for development (Conte and Walker, 2011). Additionally, to reach their final destination within the plant, iron also must be delivered to the appropriate cell compartments for utilization (Kobayashi and Nishizawa, 2012). For instance, *PIC1* and *FRO7* are required for transportation of iron to chloroplast (Duy et al., 2007; Jeong and Guerinot, 2009) and *MIT* is responsible for iron import into the mitochondrion (Bashir et al., 2011). Overall, the emerging information on iron transporters provides new insights to comprehensively understand how plants partition iron among different tissues and subcellular organelles (Kim and Guerinot, 2007).

Despite its importance, any excess of iron is highly toxic. Vacuole is a pivotal organelle for storing iron to avoid deficiency and toxicity during iron homeostasis regulation (Mendoza-Cózatl et al., 2011; Peng and Gong, 2014). Vacuolar sequestration serves as a safe iron storage strategy for detoxifying excess iron, and vacuolar iron transporters (*VITs*) were previously found to play significant roles in this process (Slavic et al., 2016). When challenged by a high iron environment, *VITs* can maintain iron within an optimal physiological range or deliver iron to enable the cell growth (Zhang et al., 2012; Narayanan et al., 2015). *VITs* help transportation of iron into vacuoles which function as buffering pools with adjustable sequestration capacity along with the environmental change to prevent cellular toxicity (Peng and Gong, 2014). In *Arabidopsis, AtVIT1* is one of the main transporters which allows iron uptake into vacuoles when exposed to iron excess (Kim et al., 2006). In contrast, *AtNRAMP3* and *AtNRAMP4* act as retrievers to export iron from vacuoles for cell growth at different stages of plant life (Lanquar et al., 2005). *AtMEB1* and *AtMEB2* are functional homologs of the yeast iron transporter *CCC1*, which serve as iron transporters to reduce the toxicity in yeast *ccc1* mutant under high iron concentration condition (Yamada et al., 2013). Moreover, two orthologs of *AtVIT1* in rice, *OsVIT1* and *OsVIT2* have been shown to modulate iron translocation between flag leaves and seeds (Zhang et al., 2012). *TgVIT1* is required for the development of blue color in the bottom of petal by controlling iron content (Momonoi et al., 2009). In addition to their roles in transporting iron, *VIT* members are poorly selective to divalent metal ions. Other potentially toxic metals can transport into vacuoles along with iron, such as Zn^2+^ and Mn^2+^. For instance, *OsVIT1* and *OsVIT2* can modulate Zn storage in the vacuole of flag leaf (Zhang et al., 2012); *AtNRAMP3* and *AtNRAMP4* help in the uptake of Mn^2+^ to photosystem II in mesophyll cells (Lanquar et al., 2010). Thus, a better understanding of *VITs* for uptake and allocation iron in plants is of great importance for iron homeostasis.

In contrast to the model plant of *Arabidopsis* and rice, relatively little is known about the contribution of *VITs* in *Brassica napus* (rapeseed). *B. napus* is an important oil crop cultivated in worldwide for providing human nutrition and oil production. Rape industry faces an urgent challenge of toxic metal pollution owing to rapid expansion and high-speed development of the industry. The mechanism of tolerance to metal toxicity in rape appears to be complex. In this report, a homolog of *AtMEB2* named as *BnMEB2* which belongs to the *VIT* family was identified in *B. napus. BnMEB2* was localized on the vacuolar membrane and participated in vacuolar sequestration of iron storage to enhance iron tolerance of the plant.

## Materials and Methods

### Plant Materials and Growth Conditions

*Brassica napus* cultivar Zhongshuang NO.9, *Arabidopsis* wild type (Col-0) and *meb2* mutant (GK-059D30, a T-DNA insertion in the second exon of *MEB2*) were used in this study. Seedlings of rapeseed cultivar Zhongshuang NO.9 were grown in 10 l boxes containing Hoagland nutrient solution in a greenhouse with 25°C light/22°C dark cycles under normal light conditions. The *Arabidopsis* wild type (Col-0), *meb2* and transgenic lines were grown on Murashige and Skoog (MS) plates containing 1% sucrose and 0.8% agar. Seedlings were grown under a 16-h-light/8-h-dark condition at 22°C in a growth chamber with light.

### Identification and Cloning of *BnMEB2*

The *B. napus* genome was downloaded from the Genoscope Genome Database^1^ (Chalhoub et al., 2014). To identify homologs in *B. napus, AtMEB2* (At5g24290) was used as initial protein queries to search against Genoscope Genome Database based on BLASTP with the score value of ≥100 and *e*-value ≤ e^-10^. Conserved VIT domain in the candidate sequences were detected by Pfam database^2^ on line. At last, two candidate genes were identified such as *BnaC07g30170D* and *BnaA06g26800D*. According to the sequence information of these two genes, specific primers (Forward 5′-ATGGACCGACCAGCTGACG-3′ and Reverse 5′-TTAAAGGGAAGTAAACCGGTATTC-3′) were designed with the Primer Premier 5.0 (PREMIER Biosoft International, USA). The coding sequence (CDS) was amplified using *B. napus* cultivar Zhongshuang NO.9 cDNA as the template and sequenced. To illustrate the structure of intron, exon and VIT domain of the candidate genes, fancygene database^3^ was used to draw the gene structure according to the full-length genome from Genoscope Genome Database and coding sequence from sequencing. The sequences of candidate genes were aligned by using Clustal W (Thompson et al., 1994; Rambaldi and Ciccarelli, 2009). The 3D protein structure model of the homologs regions was predicted by SWISS-MODEL and was analyzed by using SPDBV4.10 (Guex and Peitsch, 1997).

### Subcellular Localization of BnMEB2

Cellular localization of the BnMEB2 protein was examined by transient expression of a BnMEB2::GFP fusion protein. The open reading frames (ORFs) of *BnMEB2* without a stop codon were amplified by using the following primers (Forward 5′-GACTGGTACGAGCTCGGTACCATGGACCGACCAGCTGACG-3′; Reverse 5′-GCCCTTGCTCACCATGGATCCAAGGGAAGTAAACCGGTATTCCTC-3′). The underlined sections in primer sequences indicated *KpnI* and *BamHI* restriction sites respectively. PCR products were recombined into pUC19-35Spro::GFP (previously modified and constructed by our lab) to generate the expression vector 35Spro::BnMEB2::GFP by using ClonExpress MultiS One Step Cloning Kit (Vazyme). The detailed information about these vectors was shown in the **Supplementary Figure S1**. The resulted constructs together with the positive control 35Spro::GFP were transiently expressed in *Arabidopsis* protoplasts via polyethylene glycol-mediated transformation (Yoo et al., 2007). Furthermore, the plasmid constructs were also delivered into onion epidermal cells for transiently expression by DNA particle bombardment using the PDS-1000/He Biolistic Particle Delivery System (Bio-Rad) as described by Xie et al. (2014). GFP fluorescence was then observed with a confocal microscope (Nikon A1) at 16 h after transfection.

### *BnMEB2* Over-Expression and Plant Transformation

Due to the high similarity between *BnaC07g30170D* and *BnaA06g26800D*, only CDS fragment of *BnaC07g30170D* was constructed into pBWA(V)BS vector^4^ and verified by sequencing to create a 35Spro::BnMEB2 over-expression vector, in which target gene expression was under the control of CaMV 35S promoter. The full-length cDNAs of *BnMEB2* were amplified with the following primers (Forward 5′-CGGAATTCATGGACCGACCAGCTGACG-3′ and Reverse 5′-CGGGATCCTTAAAGGGAAGTAAACCGGTATTC-3′). The underlined sections in primer sequences indicated *EcoRI* and *BamHI* restriction sites respectively. Plasmid of 35S::BnMEB2 was introduced into *Agrobacterium tumefaciens* strain GV3101 by electroporation and was transformed into *Arabidopsis* wild type (col-0) and *meb2* mutant by floral dip method (Clough and Bent, 1998). Transgenic plants were selected by herbicide spraying. BAR-resistant and PCR-positive transgenic plants were transferred to a greenhouse and maintained up to T2 generation. Transgenic lines with high expression level of *BnMEB2* were selected and used for further analysis.

### qRT-PCR Analysis

Total RNA for quantitative RT-PCR was extracted from the rapeseed and *Arabidopsis* samples using the RNeasy Plant Minikit (Qiagen, USA) and was reverse transcribed with SuperScript^TM^ III reverse transcriptase (Invitrogen) following the manufacturer’s instructions. qRT-PCR was performed by using SYBR Green Real-time PCR Master Mix (Bio-Rad, USA) in 20 μl reaction mixture and run on CFX96 Real-time PCR system (Bio-Rad). Oilseed rape and *Arabidopsis β-actin* genes (AF111812, AT1G13180) were used as internal standard. All assays for target genes were conducted with three biological repeats, each with three technical repeats. Primers used in qRT-PCR are listed in Supplementary Table S1. The quantification of threshold cycle (CT) value analysis was achieved using the 2^(-ΔCT)^ method (Livak and Schmittgen, 2001).

### High Iron Concentration Treatment Analysis

Seeds of *B. napus* cultivar Zhongshuang NO.9 were germinated and grown in 1/2 Hoagland nutrient solution. At the eighth day, one half of the seedlings were transferred to Hoagland nutrient solution while the other half was transferred to Hoagland nutrient solution with 0.5 mM FeSO_4_ for high iron concentration treatments. Roots and leaves were collected at 1, 2, and 3 days after transferred under the normal and high iron concentration conditions and immediately frozen in liquid nitrogen. Total RNA was isolated to determine the mRNA level of *BnMEB2*. Moreover, at least three replicates of *Arabidopsis* transgenic lines (35S::*BnMEB2/WT* and 35S::*BnMEB2/meb2*), wild type (col-0) and *meb2* were grown on MS medium with a series of high iron concentration treatments (0.2-1 mM FeSO_4_) for 20 days. These seedlings were collected and their total RNAs were isolated to detect the expression level of other key *VIT* genes.

### Perls’ Staining and Iron Quantification

*Arabidopsis* roots were stained with Perls’ solution according to previous study (Green and Rogers, 2004). Equal volume of 4% (v/v) HCl and 4% (w/v) potassium ferrocyanide were mixed immediately prior to use. The mixed solution was vacuum infiltrated into 10-day-old *Arabidopsis* seedlings for approximately 15 min. Seedlings were rinsed in ultrapure water for three times. The Perls’ staining samples were observed immediately in whole roots using an Olympus IX71 microscope. The method for iron quantification was minor modified from those described by Cassin et al. (2009) and Li et al. (2013). Harvested seedlings were washed for 5 min in a solution containing 5 mM CaSO_4_ and 10 mM EDTA, then dried at 70°C for 3 days and weighted. Approximately 300 mg of dry tissue was digested completely in 10 mL 70% HNO_3_ at 200°C for 2 h. The iron contents in these samples were measured by inductively coupled plasma spectrometry SPS3000 (Seiko, Tokyo, Japan) and conducted with three biological repeats.

## Results

### *BnMEB2* Belongs to *VIT* Family

**FIGURE 1:**
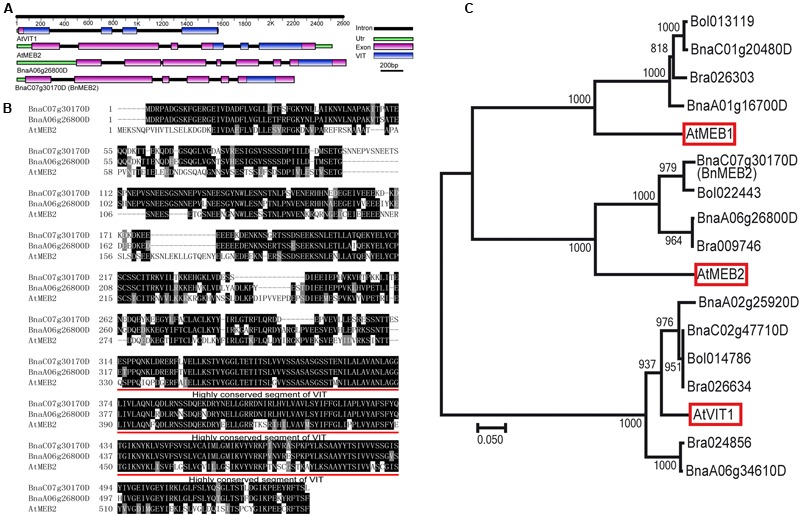
**Sequence and structure information of *BnMEB2*. (A)** Gene structural of *AtVIT1, AtMEB2* and *BnMEB2*. The different color lines and boxes represent introns, UTR, exons and VIT domain, respectively. Lengths of exons and introns of each gene are displayed proportionally. **(B)** Amino acid sequence alignment with AtMEB2. The identical amino acid residues were black-shaded and similar ones were indicated by gray-shaded. Their highly conserved sequence of VIT domains are underlined. **(C)** Phylogenetic relationship of *BnMEB2*, the vacuolar iron transporters *AtVIT1* (At2g01770), *AtMEB1* (At4g27860), *AtMEB2* (At5g24290) from *Arabidopsis thaliana* and their homologs from *Brassica rapa, Brassica oleracea* and *Brassica napus*. The scale bar represents the branch lengths.

To identify the homologs in *B. napus, AtMEB2* was used as protein queries for BLASTP and two copies (*BnaC07g30170D* and *BnaA06g26800D*) were identified from *B. napus* genome. Based on the information in Genoscope Genome Database, full-length cDNA fragments in rapeseed cultivar Zhongshuang NO.9 were amplified by high-fidelity RT-PCR. *BnaC07g30170D* locates at C07 chromosome, which consists of six exons and five introns. *BnaA06g26800D* locates at A06 chromosome, which consists of seven exons and six introns. More importantly, same as the well-known VIT family members in *Arabidopsis* such as *AtVIT1* and *AtMEB2, BnaC07g30170D* and *BnaA06g26800D* also contained VIT domain. Interestingly, VIT domains of *AtVIT1* and *AtMEB2* were separated by three or two introns but were fully integrated in the last exon of *BnaC07g30170D* and *BnaA06g26800D* (**Figure 1A**). *BnaC07g30170D* and *BnaA06g26800D* encode putative protein with 534 and 537 amino acids respectively, and show a high similarity with *AtMEB2*. Especially, VIT domain shared over 85% sequence similarity among *AtMEB2, BnaC07g30170D* and *BnaA06g26800D* (**Figure 1B**). Phylogenetic analysis indicated that *BnaC07g30170D* and *BnaA06g26800D* had a high level of similarity to these putative plant *VIT* proteins in *Arabidopsis thaliana* and *Brassiceae family* (**Figure 1C**). *BnaC07g30170D, BnaA06g26800D* and *AtMEB2* were clustered into one group and *BnaC07g30170D* was more similar to *AtMEB2* than that of *BnaC07g30170D* in the phylogenetic analysis. In addition, *BnaC07g30170D* has a higher similarity of protein sequence to *AtMEB2* than that of *BnaA06g26800D* in their highly conserved sequence of VIT domains, thus *BnaC07g30170D* was designated as *BnMEB2* for further study. Furthermore, protein 3D structure model showed that AtVIT1, AtMEB2 and BnMEB2 shared similar structures within VIT domain (**Supplementary Figure S2**). Therefore, these results suggest that *BnMEB2* belongs to the *VIT* family.

### *BnMEB2* Locates on the Vacuolar Membrane

**FIGURE 2:**
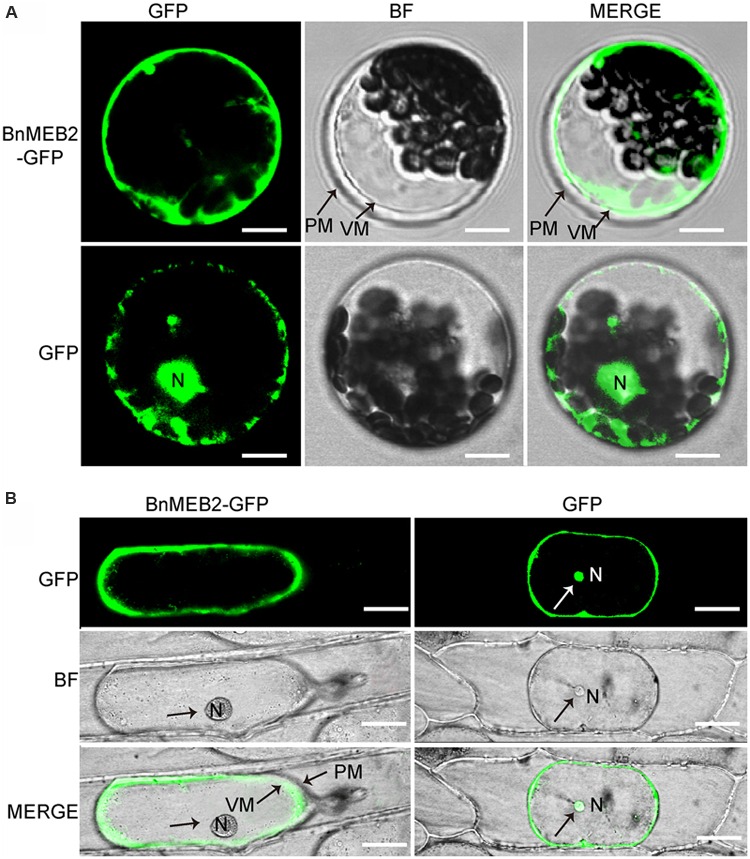
**Subcellular localization of BnMEB2. (A)** Subcellular localization of BnMEB2 in *Arabidopsis* protoplasts. Protoplasts were isolated from *Arabidopsis* seedlings and transformed with 35Spro::BnMEB2::GFP and 35Spro::GFP respectively. Bars = 10 μm. **(B)** Subcellular localization of BnMEB2 in onion epidermal cells. Bars = 20 μm. The 35Spro::GFP constructs were used as positive controls. Green fluorescence, bright field and merged images were taken under confocal. N, nucleus; VM, vacuole membrane; PM, plasma membrane.

The *VITs* were previously found to locate on vacuolar membrane to mediate vacuolar sequestration of iron storage and acted as metal transporters, such as *TgVIT1* (Momonoi et al., 2009), *AtVTL1* and *AtVTL2* (Gollhofer et al., 2014). The subcellular localization of BnMEB2 was investigated by transiently expression in *Arabidopsis* protoplasts and onion epidermal cells. BnMEB2 was fused in frame to GFP (green fluorescent protein) in the expression vector pUC19-35Spro::GFP. 35Spro::BnMEB2::GFP fusion construct and the GFP alone control 35Spro::GFP, both driven by 35S promoter were introduced into *Arabidopsis* protoplasts. BnMEB2::GFP fusion protein was observed exclusively in the vacuolar membrane while GFP alone was found throughout the control cells (**Figure 2A**). Furthermore, the subcellular localization of BnMEB2 was also investigated by expression of 35Spro::BnMEB2::GFP in onion epidermal cells. BnMEB2::GFP fluorescence was observed in the vacuolar membrane too (**Figure 2B**). The above results showed that BnMEB2 was localized on the vacuolar membrane and may also be a vacuolar membrane transporter.

### *BnMEB2* Is Highly Expressed in Mature Leaves and Can Be Induced by High Iron Concentration Treatment

**FIGURE 3:**
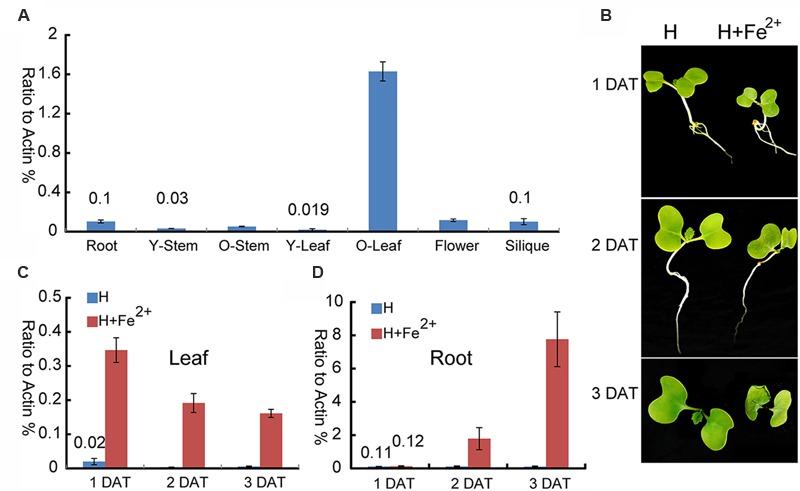
**Expression patterns of *BnMEB2* in *Brassica napus*. (A)** Relative expression levels of *BnMEB2* in *Brassica napus*. *BnActin* was used as an internal control. Root, 10-day-old roots; Y-Stem, 14-day-old stems; O-Stem, 60-day-old stems; Y-Leaf, 14-day-old leaves; O-Leaf, 60-day-old leaves; **(B)** The *Brassica napus* seedlings in response to the normal Hoagland nutrient solution **(H)** and high concentration iron treatment (H + Fe^2+^) conditions. DAT represents days after treatments. **(C,D)** The expression of *BnMEB2* in rapeseed seedling leaves and roots under normal and high concentration iron treatments. Values are the means ± SD of three independent experiments.

To provide deep insight into the function of *BnMEB2* during different physiological processes, expression pattern was examined in various tissues. Transcription levels of *BnMEB2* in all tissues of rapeseed, including root, young stem (14-day-old stem), mature stem (60-day-old stem), young leaf (14-day-old leaf), mature leaf (60-day-old leaf), flower and silique were detected by qRT-PCR (**Figure 3A**). *BnMEB2* was substantially higher expressed in mature leaves and weakly detected in other tissues including young stems and young leaves. With the storage role of leaves, this result suggested that *BnMEB2* may function and coordinate to mediate iron storage in the source tissues for human health. Interestingly, the expression of *BnMEB2* in mature stems was higher than that of young stems, and it was the same between mature and young leaves, indicating that *BnMEB2* may be highly expressed in mature tissues for advantage of iron storage during plant life cycle.

To further investigate whether *BnMEB2* is associated with iron transportation, transcriptional abundance of *BnMEB2* in *B. napus* roots and leaves were analyzed under normal Hoagland nutrient solution and high iron concentration treatment (Hoagland nutrient solution added with 500 μM FeSO_4_). The rape seedlings when treated with 500 μM FeSO_4_ showed a slow growth, yellowing of leaves and shrinking of roots when compared with the control. Three days after treatment (3 DAT), leaves of rape seedlings were obviously shrinked (**Figure 3B**). The expression of *BnMEB2* was markedly induced by high iron concentration treatment in both roots and leaves. The transcription level of *BnMEB2* was up-regulated at least 10-fold after 2 days treatment, and more than 70-fold for 3 days after treatment in seedling roots (**Figure 3C**). Similar results were obtained in leaves, where significant induction of *BnMEB2* was observed after 1, 2, and 3 days of high iron concentration treatment (**Figure 3D**). Furthermore, in comparison with roots, the induction of *BnMEB2* expression was faster in young leaves. In young leaves, at the first day after high iron concentration treatment did *BnMEB2* show remarkably higher expression than the second and third days under the same treatment. This phenomenon might be responsible for iron storage in leaves to detoxify the iron stress, while roots are mainly responsible for iron acquisition. Moreover, the expression of *BnMEB2* was higher in young roots than in young leaves (**Figures 3C,D**). Taken together, these data imply that *BnMEB2* was highly induced by high iron concentration treatment and was involved in iron transportation.

### Over-Expression of *BnMEB2* Enhances the Tolerance of High Iron Concentration stress in *Arabidopsis* Transgenic Plants

**FIGURE 4:**
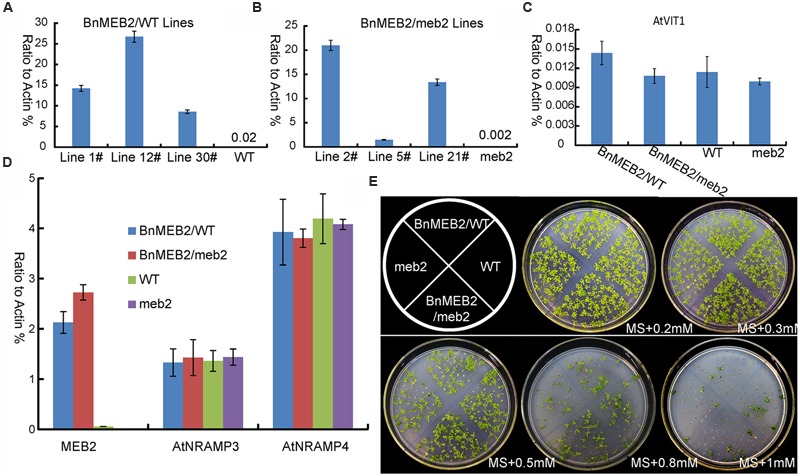
**Improved iron tolerance and the expression of vacuoleiron transporter genes in transgenic *Arabidopsis*. (A,B)** Expression level of *MEB2* in *BnMEB2* over-expression in *Arabidopsis* wild type (WT, col-0) background (*35S::BnMEB2/WT*) and mutant *meb2* (*35S::BnMEB2/meb2*) background transgenic lines. Seedlings were grown for 30 days and total RNAs were extracted for quantitative RT-PCR analysis. **(C,D)** The expression level of iron transporter genes in WT, *meb2* and transgenic *Arabidopsis*. Twenty-day-old seedlings of WT, *meb2, 35S::BnMEB2/WT* and *35S::BnMEB2/meb2* which grown on MS medium were treated with FeSO_4_ (0.5 mM). *AtActin* was used as an internal control. **(E)** The comparison analysis of iron tolerance in *Arabidopsis* transgenic plants. The seedlings of WT, *meb2, 35S::BnMEB2/WT* and *35S::BnMEB2/meb2* were cultured on MS agar medium with a series of iron concentration (0.2 mM, 0.3 mM, 0.5 mM, 0.8 mM and 1 mM FeSO_4_) for 20 days.

To better understand the role of *BnMEB2* in regulation the tolerance of high iron concentration stress, *BnMEB2* over-expression vector driven by 35S promoter was constructed and transformed into *Arabidopsis* wild type (col-0) and *meb2* (defined as 35S::*BnMEB2/WT* and 35S::*BnMEB2/meb2*, respectively). A total of three 35S::*BnMEB2/WT* (line 1#, 12#, and 30#) and three 35S::*BnMEB2/meb2* (line 2#, 5#, and 21#) transgenic lines were selected and purified. qRT-PCR analysis showed that the expression levels of *BnMEB2* in transgenic plants with either WT or *meb2* background were significantly increased as compared with wild type and *meb2* mutant (**Figures 4A,B**). To verify the functions of *BnMEB2 in vivo*, these transgenic lines were challenged with high iron concentration stress. 35S::*BnMEB2/WT* transgenic line 1#, 35S::*BnMEB2/meb2* transgenic line 21#, wild type and *meb2* were sowed on MS medium under a series of high iron concentration conditions (0.2 mM, 0.3 mM, 0.5 mM, 0.8 mM and 1 mM FeSO_4_) and cultured for 20 days. As shown in **Figure 4E**, the high iron concentration significantly impacted on seeds germination at the seedling stage. 35S::*BnMEB2/WT* and 35S::*BnMEB2/meb2* transgenic plants had a higher seeds germination rate and more tolerated to high iron environment than that of *meb2*, especially when the high iron concentration increasing to more than 0.5 mM FeSO_4_. Over-expression of *BnMEB2* restored and increased the germination rate of *meb2* in the complementary lines 35S::*BnMEB2/meb2*. These results showed that 35S::*BnMEB2/WT* and 35S::*BnMEB2/meb2* transgenic plants had stronger high iron tolerance than that of *meb2*, indicating that *BnMEB2* was involved in high iron tolerance.

The enhanced tolerance of *BnMEB2* over-expression plants to iron stress prompts us to evaluate the expression level of other key *VIT* genes such as *AtVIT1, AtNRAMP3* and *AtNRAMP4* in all four above lines. It was previously revealed that *AtVIT1* functioned as the main transporter allowing iron uptake into vacuoles when exposed to excess iron (Kim et al., 2006). *AtNRAMP3* and *AtNRAMP4* genes specifically acted as a retriever to export iron from vacuoles when at the deficient iron conditions (Lanquar et al., 2005). The transcription level of *AtVIT1,AtNRAMP3* and *AtNRAMP4* showed no significant differences among *35S*::*BnMEB2* transgenic lines and *meb2* when compared with wild type plants (**Figures 4C,D**), while the expression of *MEB2* gene was dramatically increased in the 35S::*BnMEB2/WT* and 35S::*BnMEB2/meb2* transgenic plants and strongly suppressed in *meb2* (**Figure 4A**). These results suggested that the enhanced iron tolerance in 35S::*BnMEB2/WT* and 35S::*BnMEB2/meb2* transgenic plants was mainly caused by the high expression of *BnMEB2*. Over-expression of *BnMEB2* restored the iron tolerance ability of *meb2* in the complementary lines, indicating that abundant expression of *BnMEB2* facilitated the iron storage in plant vacuoles and thus leading to better tolerate the high iron concentration stress. Hence, *BnMEB2* plays important roles in the tolerance of high iron concentration stress.

### *BnMEB2* Increases Plant Detoxification Ability of Iron Stress

**FIGURE 5:**
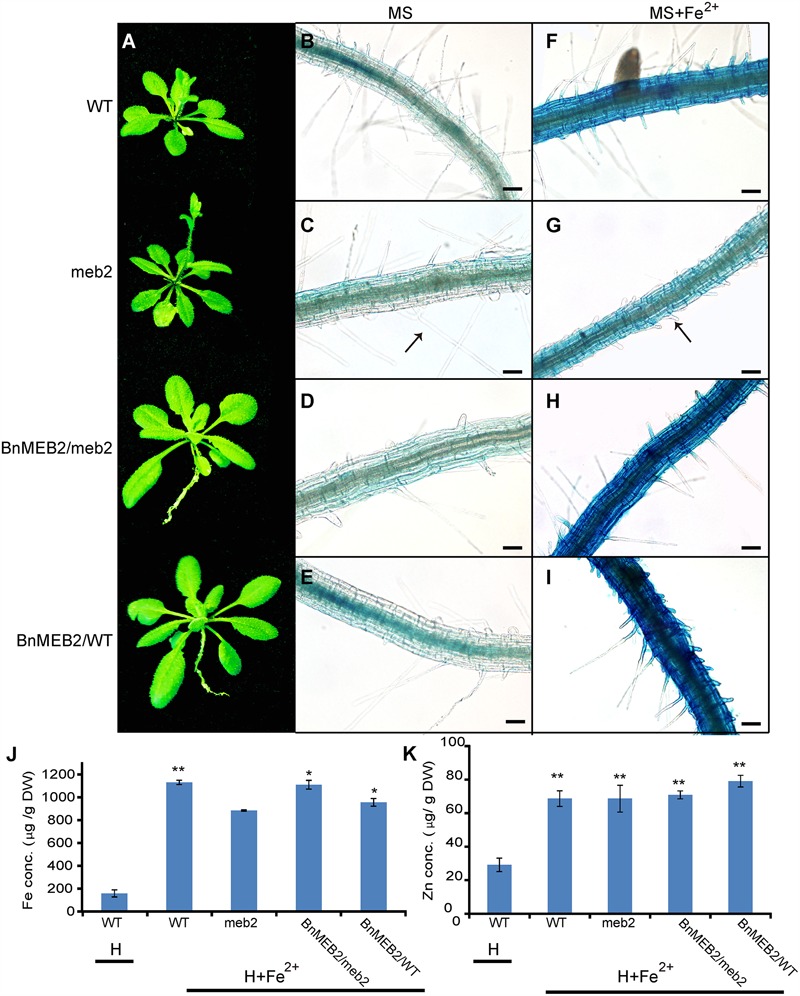
**The detoxification ability of *BnMEB2.***(A)** Phenotypes of WT, *meb2* and transgenic *Arabidopsis*.** Three-week-old seedlings of WT, *meb2, 35S::BnMEB2/WT* and *35S::BnMEB2/meb2* were grown in 0.5 mM FeSO_4_ iron nutrient solution (H+Fe^2+^). **(B–I)** The iron storage in roots of WT, *meb2* and transgenic *Arabidopsis* by representative Perls’ staining. The seedlings of WT, *meb2, 35S::BnMEB2/WT* and *35S::BnMEB2/meb2* were grown on MS medium **(B–E)** and MS medium added with 0.5 mM FeSO_4_ **(G–I)** for 10 days. The arrow indicates the root hairs. The bar means 20 μm. **(J,K)** Iron contents and zinc contents in the seedlings of WT, *meb2* and transgenic *Arabidopsis*. The seedlings of WT, *meb2, 35S::BnMEB2/WT* and *35S::BnMEB2/meb2* were grown in 0.5 mM FeSO_4_ iron nutrient solution (H + Fe^2+^) for 30 days. Another set of the WT seedlings were grown in the normal Hoagland nutrient solution (H) for 30 days. Both sets incubated in the same environment conditions in a growth room. Statistical analysis were performed between WT under the H condition and each of four genotypes under the H + Fe^2+^ condition. Data are mean ± SD, ^∗^*P* < 0.05, ^∗∗^*P* < 0.01.

To deeply characterize the mechanism of enhanced iron tolerance in *BnMEB2* over-expressed *Arabidopsis* transgenic plants, iron storage in roots of WT, *meb2*, 35S::*BnMEB2/WT* and 35S::*BnMEB2/meb2* plants were determined by representative Perls’ staining. Iron and zinc contents in these seedlings were also measured by using inductively coupled plasma spectrometry. Under high iron concentration condition (0.5 mM FeSO_4_), 35S::*BnMEB2/WT* and 35S::*BnMEB2/meb2* transgenic plants showed a better growth status with bigger rosette leaves and developed root system when compared with *meb2* (**Figure 5A**). Iron localization in roots were very similar among WT, *meb2* and *35S::BnMEB2/meb2*, only a slight higher in *35S::BnMEB2/WT* when grown on MS medium (**Figures 5B–E**). While under the MS medium with 0.5 mM FeSO_4_ condition, distinct blue staining was observed and became intensified in the roots of WT, *35S::BnMEB2/meb2* and *35S::BnMEB2/WT* transgenic plants (**Figures 5F–I**). However, iron distribution in roots of *meb2* had a little alternation between normal and high iron concentration conditions but root hairs elongation was obviously inhibited under iron stress condition (**Figures 5C,G**). Furthermore, when challenged by high iron concentration treatment, the iron and zinc contents were significantly increased in all types plants when compared with the WT under normal condition (**Figures 5J,K**). Interestingly, with high iron concentration treatment, iron contents in the seedlings of *35S::BnMEB2/meb2, 35S::BnMEB2/WT* transgenic plants and WT were significantly higher than that of *meb2* (**Figure 5J**), but zinc contents in all four seedlings were similar with each other (**Figure 5K**). These results indicated that over-expression of *BnMEB2* increased iron storage in the vacuole of root cells and iron content in the *Arabidopsis* seedlings in response to high iron concentration stress, resulting in increased detoxification ability in *BnMEB2* transgenic plants which ensured the normal growth and development.

## Discussion

### *BnMEB2* Is a Novel Vacuolar Membrane Located *VIT* Member

Over the past decade, the important biological functions of several *VITs* in *Arabidopsis*, rice and other plant species had been characterized as vacuolar sequestration of iron storage, such as *AtVIT1, OsVIT1, OsVIT2* and *TgVIT1*(Kim et al., 2006; Momonoi et al., 2009; Zhang et al., 2012). In *Arabidopsis, AtVTL1, AtVTL2* and *AtVTL5* which showed significant homology to *AtVIT1*, also catalyzed transportation of iron into vacuoles and thus contributed to the regulation of iron homeostasis in the plant (Gollhofer et al., 2014). Another protein *AtMEB2* was a vital member of *VIT* family, and located on endoplasmic reticulum (ER) body membrane involving in iron homeostasis (Yoshida and Negishi, 2013). In general, proteins containing the conserved iron transportation motif of VIT domain are assigned to the *VIT* family, being responsible for the regulation of cytosolic iron homeostasis (Yoshida and Negishi, 2013). In this study, we identified a homolog of *AtMEB2* in *B. napus* and assigned as *BnMEB2*. *BnMEB2* also contained a VIT domain in the C-terminal region (**Figure 1A**). By clustal and phylogenetic analysis, *BnMEB2* and *BnaA06g26800D* shared a high similarity with *AtMEB2* and also shared similar 3D structures of VIT domain with AtVIT1 and AtMEB2 (**Figures 1B,C**; **Supplementary Figure S2**). Notably, it is peculiar that VIT family members were located on the vacuolar membrane. BnMEB2 also was located on vacuolar membrane (**Figures 2A,B**), indicating a conservative vacuolar metal transport function for *BnMEB2*. Therefore, it is reasonable to propose that *BnMEB2* is a novel *VIT* member with underlying ability of metal iron transportation.

### *BnMEB2* Enhances Iron Tolerance by Participating in Iron Storage for Vacuolar Detoxification in Plant

Incapable for escaping from unwanted changes in the environment, the sessile plants have developed a series of tolerance strategies such as vacuolar sequestration, metal chelation, and metal eﬄuxion to cope with the negative consequences of metal toxicity (Clemens, 2006; Singh et al., 2016). *AtMEB2* was involved in the metal ion homeostasis and enhanced iron resistance in yeast *ccc1* mutant (Yamada et al., 2013). In *B. napus* seedlings, expression of *BnMEB2* was strongly induced under high iron concentration treatment in both roots and leaves (**Figures 3C,D**), suggesting that *BnMEB2* might play important role in iron homeostasis when plants grew under such conditions. At the stages of germination and growth, over-expression *BnMEB2* transgenic seedlings had stronger iron tolerance while the seedlings of *meb2* were more sensitive to high iron concentration stress (**Figure 4E**). The expression of *MEB2* gene was dramatically up-regulated while other key *VIT* genes showed no significant differences among them (**Figures 4A–D**). These observations lead to the conclusion that the alteration of iron tolerance is closely related to the expression level of *BnMEB2*.

As far as metal toxicity is concerned, the most important mechanism of metal tolerance in plants is vacuolar detoxification. Although iron can be stored in ferritin protein in chloroplast and mitochondria, vacuolar detoxification serves as a principal and safe strategy in iron storage. In *Arabidopsis*, only 5% of iron was incorporated in ferritin protein, while vacuoles appear to be the major compartment for iron storage (Ravet et al., 2009). *BnMEB2* did not affect the root hair development when grown on MS medium (**Figures 5B–E**). However, root hair elongation was obviously restrained in *meb2* and iron content of *meb2* was lower when compared with other seedlings under 0.5 mM FeSO_4_ condition (**Figures 5G,J**), suggesting that loss function of *MEB2* resulted in iron toxicity of roots cell and affect roots development which in turn to decrease iron acquisition. On the contrary, over-expression *BnMEB2* transgenic seedlings were grown and developed normally even if iron distribution and acquisition significantly increased about five folds (**Figures 5F–I**). Simultaneously, BnMEB2 located on vacuolar membrane (**Figure 2**) and the expression level of *BnMEB2* was critical for iron tolerance, showing that the high expression level of *BnMEB2* acted as a vacuolar sequestration of iron storage to detoxify iron stress. Zinc contents in all types of plants increased from about 30–70 μg/g DW (dry weight), while no significant differences were detected among each other under low zinc concentration condition (**Figure 5K**), indicating that the increasing of one metal ion might activate roots to acquire other divalent metal too which further harden toxic metal stress, such as over-expression of *NAS* causing to concomitant increasing in iron and zinc content in rice grains (Johnson et al., 2011). Thus, *BnMEB2* enhances iron tolerance which significantly plays a role in plants adapting to adverse environment.

### *BnMEB2* Sheds a New Light on Homeostasis Regulations in Iron Toxicity and Tolerance

**FIGURE 6:**
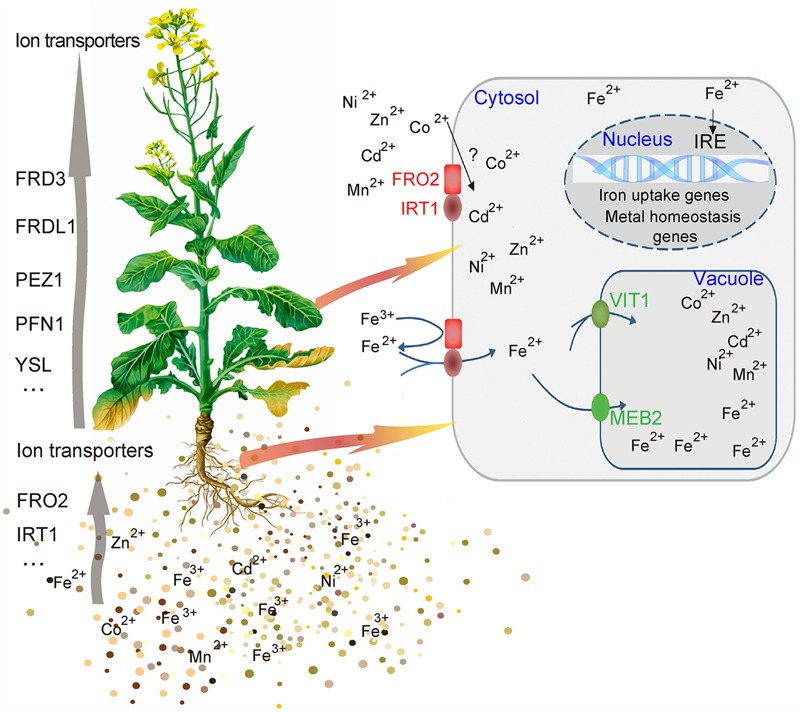
**Potential signaling of iron homeostasis.** A hypothetical model was shown the mechanism of *BnMEB2* enhancing the iron tolerance in *Brassica napus*.

The complex process of iron homeostasis must be highly coordinated by iron uptake, long-distance transport and distribution to different tissues and cell compartments. In the process of iron transport in plant, the *VIT* members contribute to iron trafficking in vacuoles. Vacuoles are the crucial compartment for storage and detoxification of iron excess and a buffering pool for iron remobilization in periods of iron deficiency. Based on the results above, this study showed that BnMEB2 was involved in high iron tolerance of *B. napus*. Thus, it is reasonable to propose a model for *BnMEB2* regulation in iron homeostasis and enhancing iron tolerance in *B. napus*, which are partially schematically represented in **Figure 6**. When challenged by a high level of iron environment, abundant Fe^3+^ is firstly reduced to more soluble Fe^2+^ by *FRO2* and is acquired into the root cells with poorly selective by membrane transporter *IRT1*, causing to iron excess and toxic in cytoplasm of cell. The status of iron excess triggers a significant up-regulated expression of iron transporter genes such as *BnMEB2*. Then, vacuolar membrane located *BnMEB2* traffics of iron into the vacuoles for storage and to avoid iron toxicity. Finally, the iron level in cytoplasm rebalances within an optimal physiological range and the plants result in more tolerant to high iron concentration stress. Briefly, *BnMEB2* enhances iron tolerance by the vacuolar sequestration and vacuolar detoxification strategy. It provides a new insight on iron homeostasis in the tolerance of high iron concentration stress.

### *BnMEB2* Highlights a Potential Strategy for Iron Biofortification and Human Health

With the tremendous rise of industrialization emissions of toxic metals, the increasing serious toxic metal pollution strongly interferes with the crops metabolism, development, and thus productivity and yield (Lutts and Lefèvre, 2015). The *VIT* members play important roles in maintaining the metal ion availability in the cytosol within an optimal range by appropriate sequestration into or retrieval from the vacuoles. For instance, over-expression of *AtPCS1* in tobacco activated high levels of phytochelatin synthesis to increase detoxification capacity to toxic metal stress (Zanella et al., 2016). Over-expression of *AtVIT1* increased accumulation of iron in the edible part of storage roots and stems of cassava (Narayanan et al., 2015). This study finds *BnMEB2* conservatively localizing on the vacuolar membrane and enhancing tolerance to high iron concentration stress. Therefore, over-expression of *BnMEB2* can be applied to minimize the serious threat of metal toxic stress and improve the survival and yield of *B. napus* and other crops. The current study highlights the significance of *BnMEB2* potential application.

Iron is a required micro-nutrient for all living organisms. Iron deficiency anemia is one of the most prevalent micro-nutrient deficiencies in the world, affecting almost 25% of the world population (Clemens et al., 2002; Cvitanich et al., 2010). Thus, increasing iron biofortification in vegetable crops of *Brassica* family could have a positive significance on human health. In higher plants, to maintain the structural and functional integrity of thylakoid membranes in chloroplasts (Kim and Guerinot, 2007), approximately 90% of iron abundance is required to allocate in the leaves (Terry and Abadía, 1986). When *B. napus* was challenged by high iron environment, considerable iron amounts were accumulated in stomata guard cells of leaves (Tewari et al., 2013). Meanwhile, in oilseed rape mature leaves, *BnMEB2* is highly expressed and can be induced by iron stress which revealed a close relationship between *BnMEB2* and iron storage (**Figure 3A**). In our previous study, *Bol022443* and *Bra009746*, significant homologies to *AtMEB2* were identified in *B. rapa* and *B. oleracea* respectively. They are highly conserved in evolution and all strongly expressed in leaves by previous trancriptome detection (Tong et al., 2013; Liu et al., 2014; Zhou et al., 2014), suggesting that *MEB2* was involved in iron distribution and storage in *Brassicaceae* leaves. For *Brassica* vegetables, their leaves are the main edible source for supplying vitamins, cellulose, minerals and other kinds of nutrition for human health. Study on *MEB2* sheds light on a potential strategy for iron biofortification in *Brassica* crops.

## Conclusion

Vacuolar detoxification is the key mechanism to tolerate metal toxic stress in plant cells, which resulted in the removal of toxic ions from sensitive sites to vacuolar compartments by various *VIT* members (Sharma et al., 2016). Enhanced iron tolerance by vacuolar detoxification is a safe strategy for plant to survive and adapt to adverse environmental conditions. In this research, a novel functional gene *BnMEB2* in rapeseed plant was proposed in vacuolar detoxification and iron tolerance. *BnMEB2* is a vacuolar membrane located new *VIT* gene which was highly expressed in mature leaves and can be strongly induced by high iron concentration stress in the roots and leaves. The transcript in level of *BnMEB2* is closely related to tolerance of plants under high iron concentration stress. *BnMEB2* is the first identified *VIT* gene in rapeseed with vacuolar detoxification function, acting as a *VIT* to mediate iron storage into vacuoles to detoxify iron stress and finally enhancing the tolerance to high iron concentration stress. These findings obtained in the study can be useful for the genetic improvement of iron tolerance and improve the nutrition value in *Brassica* crops breeding.

## Author Contributions

WZ, CD, and SL conceived the idea, designed the experiments and wrote the manuscript. WZ, RZ, and MT performed the experiments. XC, YL, and YX contributed materials and analysis tools to this work. RFZ, CT, and JH analyzed the data. All the authors approved the final manuscript.

## Conflict of Interest Statement

The authors declare that the research was conducted in the absence of any commercial or financial relationships that could be construed as a potential conflict of interest.

## Footnotes

1 http://www.genoscope.cns.fr/brassicanapus/

2 http://pfam.xfam.org/

3 http://bio.ieo.eu/fancygene/

4 http://www.biorun.net/

## Supplementary Material

FIGURE S1 **The diagram of pUC19-35Spro::GFP vector **(A)** and 35Spro::BnMEB2::GFP vector **(B)** which showed detailed information**.

FIGURE S2 **Comparison analysis of 3D structure model of VIT domain in AtVIT1, AtMEB2 and BnMEB2.** The green, red and blue bands indicate AtVIT1, AtMEB2 and BnMEB2, respectively. **(A)** BnMEB2 versus AtVIT1; **(B)** BnMEB2 versus AtMEB2.
