# Supplementary material for: Vacuolar Iron Transporter BnMEB2 Is Involved in Enhancing Iron Tolerance of Brassica napus
Source: Front Plant Sci. 2016 Sep 13;7:1353. doi: 10.3389/fpls.2016.01353 (PMC5020681; doi:10.3389/fpls.2016.01353)
Supplement: Supplementary file 1 [file Table_1.DOCX]

**Supplementary Table S1. Primers used in qRT-PCR**

| **Genes** | **Forward Primers (5' to 3')** | **Reverse Primers (5' to 3')** |
| --- | --- | --- |
| **BnActin** | **CTGGAATTGCTGACCGTATGAG** | **ATCTGTTGGAAAGTGCTGAGGG** |
| **MEB2** | **CGTGCATCCCCTAAACCTTA** | **TCTCCCACGATTTCTCCAAC** |
| **AtActin** | **CCCGCTATGTATGTCGCCA** | **AACCCTCGTAGATTGGCACA** |
| **AtVIT1** | **GTTCCTGAAACTGAGGCAGC** | **CAAGCCAAGCTTGAGGATTC** |
| **AtNRAMP3** | **GATCGGGCTATTAGCTGCTG** | **AGCACGTTAAGCCACTCGTT** |
| **AtNRAMP4** | **GCACTTGTGCAATCCAGAGA** | **GAAAGAAACCGCAAGAGCAC** |
