# Supplementary figures and images for: Vacuolar Iron Transporter BnMEB2 Is Involved in Enhancing Iron Tolerance of Brassica napus
Source: Front Plant Sci. 2016 Sep 13;7:1353. doi: 10.3389/fpls.2016.01353 (PMC5020681; doi:10.3389/fpls.2016.01353)

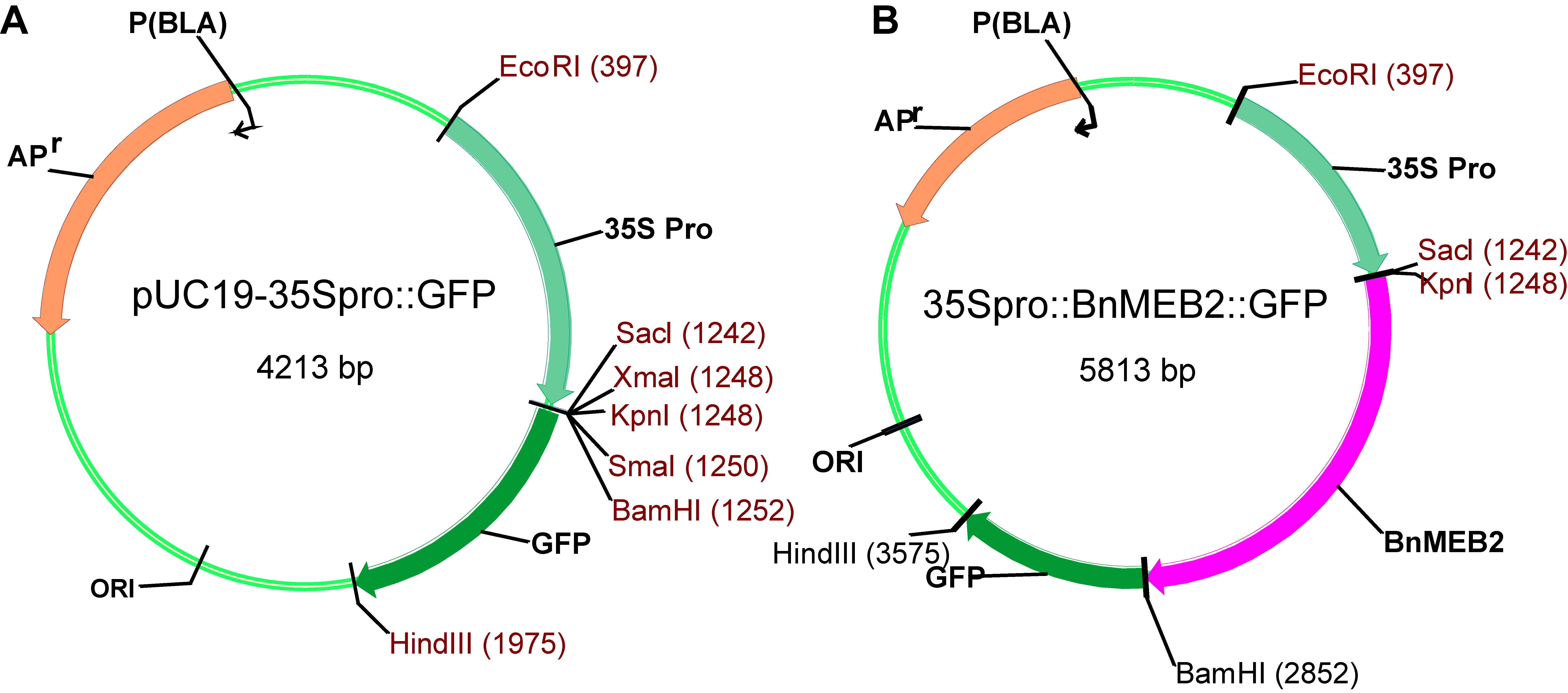

Supplement: FIGURE S1 — The diagram of pUC19-35Spro::GFP vector (A) and 35Spro::BnMEB2::GFP vector (B) which showed detailed information. [file Image_1.TIF]

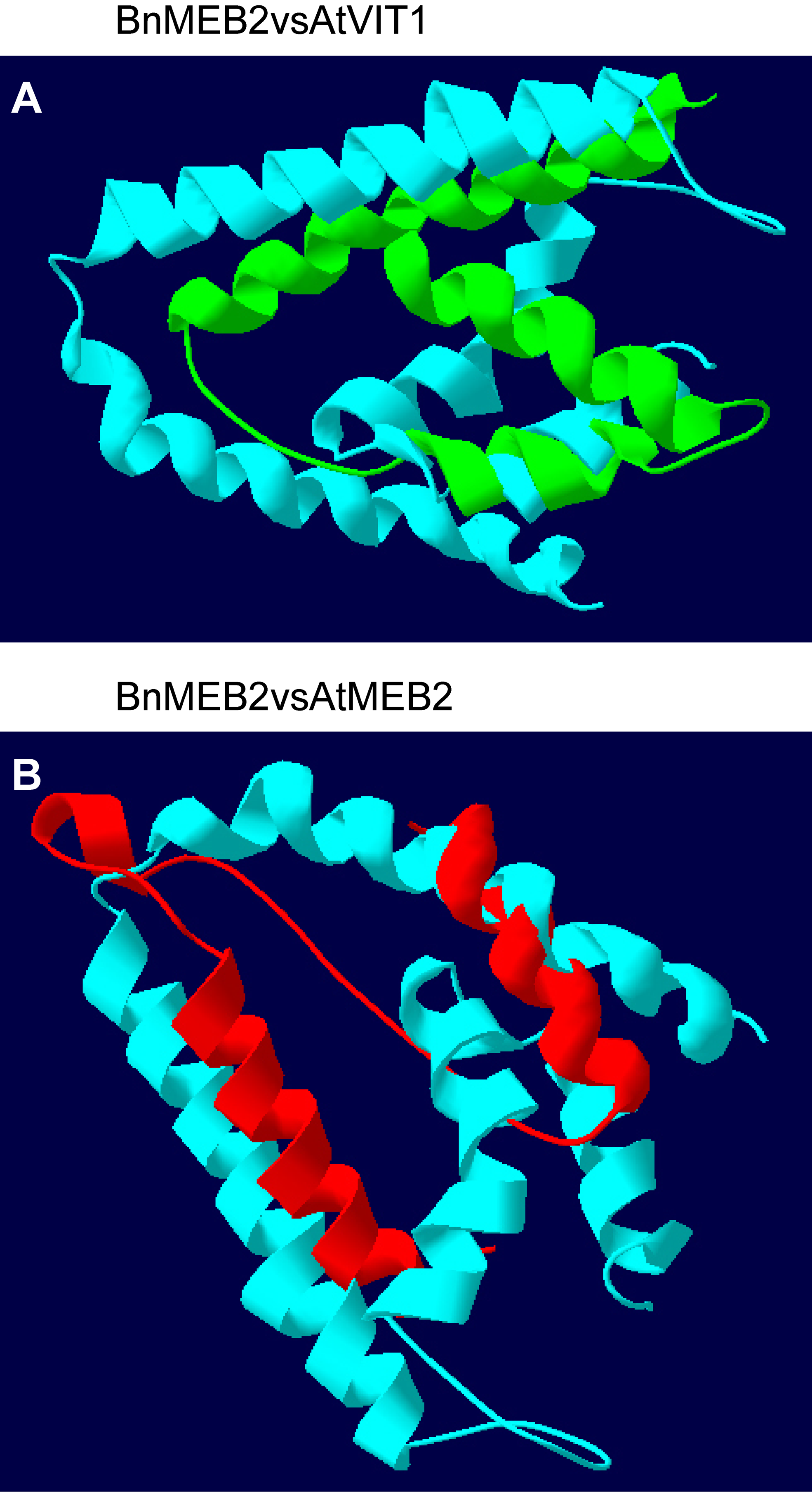

Supplement: FIGURE S2 — Comparison analysis of 3D structure model of VIT domain in AtVIT1, AtMEB2 and BnMEB2. The green, red and blue bands indicate AtVIT1, AtMEB2 and BnMEB2, respectively. (A) BnMEB2 versus AtVIT1; (B) BnMEB2 versus AtMEB2. [file Image_2.TIF]
